# The Liver-Stage *Plasmodium* Infection Is a Critical Checkpoint for Development of Experimental Cerebral Malaria

**DOI:** 10.3389/fimmu.2019.02554

**Published:** 2019-11-01

**Authors:** Yuko Sato, Stefanie Ries, Werner Stenzel, Simon Fillatreau, Kai Matuschewski

**Affiliations:** ^1^Parasitology Unit, Max Planck Institute for Infection Biology, Berlin, Germany; ^2^Department of Microbiology and Immunology, Keio University School of Medicine, Tokyo, Japan; ^3^Immune Regulation Research Group, Deutsches Rheuma-Forschungszentrum, Berlin, Germany; ^4^Department of Neuropathology, Charité - Universitätmedizin, Freie Universität Berlin, Humboldt-Universität zu Berlin, and Berlin Institute of Health (BIH), Berlin, Germany; ^5^Department of Immunology, Infectiology and Haematology (I2H), Institut Necker-Enfants Malades, INSERM U1151-CNRS UMR 8253, Paris, France; ^6^Faculté de Médecine, Université Paris Descartes, Sorbonne Paris Cité, Paris, France; ^7^AP-HP, Hôpital Necker Enfants Malades, Paris, France; ^8^Department of Molecular Parasitology, Institute of Biology, Humboldt University, Berlin, Germany

**Keywords:** *Plasmodium*, malaria, cerebral malaria, experimental cerebral malaria, liver-stage, pre-erythrocytic stage, sporozoites

## Abstract

Cerebral malaria is a life-threatening complication of malaria in humans, and the underlying pathogenic mechanisms are widely analyzed in a murine model of experimental cerebral malaria (ECM). Here, we show abrogation of ECM by hemocoel sporozoite-induced infection of a transgenic *Plasmodium berghei* line that overexpresses profilin, whereas these parasites remain fully virulent in transfusion-mediated blood infection. We, thus, demonstrate the importance of the clinically silent liver-stage infection for modulating the onset of ECM. Even though both parasites triggered comparable splenic immune cell expansion and accumulation of antigen-experienced CD8^+^ T cells in the brain, infection with transgenic sporozoites did not lead to cerebral vascular damages and suppressed the recruitment of overall lymphocyte populations. Strikingly, infection with the transgenic strain led to maintenance of CD115^+^Ly6C^+^ monocytes, which disappear in infected animals prone to ECM. An early induction of IL-10, IL-12p70, IL-6, and TNF at the time when parasites emerge from the liver might lead to a diminished induction of hepatic immunity. Collectively, our study reveals the essential role of early host interactions in the liver that may dampen the subsequent pro-inflammatory immune responses and influence the occurrence of ECM, highlighting a novel checkpoint in this fatal pathology.

## Introduction

One of the most severe pathological complications caused by *P. falciparum* infection is cerebral malaria (CM) (1, 2). As an experimental murine model for CM, *P. berghei* ANKA infection of C57BL/6 mice with a Th1-biased phenotype is well-established and termed experimental cerebral malaria (ECM) (3). The ECM model recapitulates many aspects of human pathology, such as up-regulation of inflammatory cytokines, activation of cerebral endothelial cells, platelet accumulation, sequestration of leukocytes and infected red blood cells (iRBCs), reduced blood flow, intracranial hypertension and hemorrhages, which together lead to irreversible fatal cerebral pathology (4–10). ECM results in rapid death often occurring within 4–5 h after the onset of the first neurological signs, including ataxia, respiratory distress, seizure, and coma (11, 12).

A central hallmark of ECM is destruction of the blood-brain barrier (BBB) (12). It is now well-established that cytotoxic CD8^+^ T cells are the primary mediators of ECM development (13–21). During ECM, parasite-specific CD8^+^ T cells accumulate along cerebral vessels, where INF-γ release is thought to cause the activation of endothelial cells and perforin-mediated disruption of tight junctions to induce the BBB breakdown (20–24).

A major research focus of ECM has been on terminal immune responses that take place in the brain using blood transfusion of infected red blood cells, which have immensely advanced our understanding of the underlying mechanisms of ECM pathogenesis. However, there is very limited information on how the pre-erythrocytic phase of an infection can influence the disease outcome, where sporozoites from infectious *Anopheles* mosquitoes are inoculated, followed by parasite propagation in the host liver. Currently, the vast majority of ECM research is conducted by bypassing the pre-erythrocytic phase and directly starts the experiments from blood-stage infections.

In this study, we investigated the pathogenesis of ECM in C57BL/6 mice using transgenic *Plasmodium berghei* ANKA parasites that moderately over-express profilin under the control of the *apical membrane antigen 1* (*AMA1*) promoter (25). Profilin is essential for blood infection and is likely involved in nucleotide re-loading of actin monomers, thus accelerating the microfilament turnover and modulating parasite motility (26, 27). Transgenic parasites, termed PRF parasites herein, express elevated (~10-fold increase) levels of profilin in sporozoites and blood-stage parasites (25). All experiments were conducted with hemocoel sporozoites, since they display similar virulence and immunogenicity as sporozoites isolated from salivary glands (28), and PRF sporozoites display decreased salivary gland invasion (25). Lower salivary gland infectivity is in good agreement with premature sporozoite maturation and led us to hypothesize that PRF sporozoites might display enhanced liver infection and population expansion, which has not yet been achieved by experimental genetics.

We show that PRF blood stages are fully virulent and pathogenic, since infections with asexual blood-stage parasites cause ECM. However, these parasites are unable to elicit ECM pathology when the infection is induced by sporozoites. Remarkably, this abrogation of ECM occurred upon comparable onset of blood-stage development, thus allowing us to study, for the first time, how the liver phase affects the subsequent development of the cellular immune responses ultimately leading to ECM. Collectively, our data reveal the pre-erythrocytic phase of infection as a novel checkpoint for the development of the subsequent immune response and the progression to fatal immunopathology.

## Results

### Enhanced Transmigration and Invasion of Hepatocytes by Transgenic PRF Sporozoites

**Figure 1:**
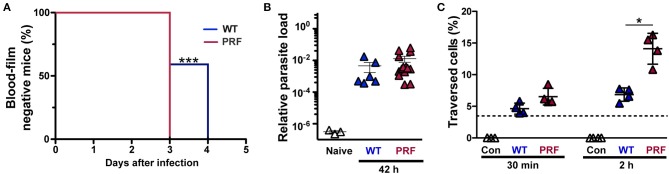
Improved pre-erythrocytic development of PRF parasites. **(A)** Prepatent period of sporozoite-induced infections. Appearance of blood-stage parasites was monitored by daily microscopic examination of Giemsa-stained blood films. C57BL/6 mice were infected by intravenous injection of 5,000 WT or PRF hemocoel sporozoites (*n* = 22 each). Shown is a Kaplan-Maier analysis of time to first detection of blood infection, ^***^P < 0.001 (Mantel-Cox test). **(B)** *In vivo* quantification of parasite loads in the liver of infected mice. Livers were harvested 42 h after infection of C57BL/6 mice by intravenous injection of 5,000 WT or PRF hemocoel sporozoites. Expression levels of *P. berghei* 18S rRNA were quantified by real-time RT-PCR and normalized to mouse *GAPDH*. Results represent mean values (± SEM) (*n* = 8 each for infected mice; *n* = 3 for naïve mice). Differences between WT- and PRF-infected livers were non-significant (Mann-Whitney test). **(C)** Sporozoite cell traversal. Hepatoma cells were incubated for 30 min or 2 h with medium (white; Con), FITC-dextran only (dotted line), and FITC-dextran together with either WT (blue; WT), or PRF (red; PRF) hemocoel sporozoites. Cells were analyzed by flow cytometry to enumerate the percentage of dextran-positive cells indicative of sporozoite traversal. Results represent mean values (±SD) of at least three independent experiments with duplicates each. ^*^P < 0.05 (Mann-Whitney test).

We first characterized pre-erythrocytic development of PRF sporozoites and injected susceptible mice with 5,000 freshly dissected hemocoel sporozoites from wild-type (WT)- or PRF-infected *Anopheles stephensi* mosquitoes (Figure 1A). The prepatent period was 3 days for all mice infected with PRF sporozoites, whereas mice infected by WT sporozoites required up to 3–4 days for microscopic detection of blood-stage parasites by Giemsa-stained blood smears (Figure 1A). To gain a better understanding of liver-stage infection by PRF parasites *in vivo* and *in vitro* experiments were conducted. Quantification of the parasite load in the liver 42 h after inoculation with sporozoites revealed no difference between WT or PRF parasites (Figure 1B), suggesting full maturation of PRF liver stages. However, when PRF sporozoites were deposited onto cultured hepatoma cells for 2 h, we detected elevated levels of transmigration in comparison to WT sporozoites (Figure 1C). Enumeration of liver stages in cultured hepatoma cells revealed higher numbers for PRF infection compared to WT infection after 24 and 48 h (Supplemental Figure 1).

Together, PRF sporozoites are enhanced in cellular attachment (25), as well as transmigration and invasion of liver cells as compared to WT sporozoites. This gain of function is unprecedented and allowed us to explore, whether enhancement of the first parasite—host interactions may lead to modulations of infection and immune responses.

### Absence of ECM Pathology After Infection With PRF Sporozoites but Not Blood Transfusion

**Figure 2:**
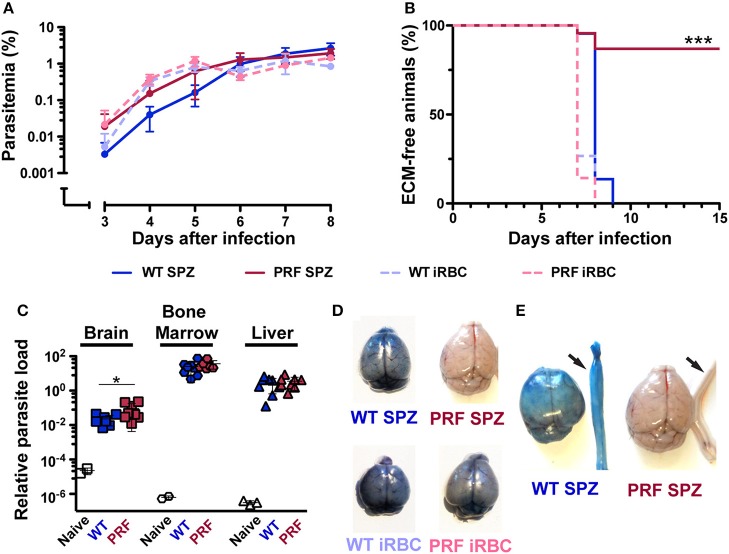
Full virulence after PRF blood infection, but very low ECM pathology after sporozoite infection. **(A)** Time course of blood infection after sporozoite inoculation or transfusion of iRBCs. C57BL/6 mice were infected by intravenous injection of 5,000 hemocoel sporozoites (solid lines) of either wild type (WT; blue) or PRF (red) parasites (*n* = 22 each) or 5,000 iRBCs (dashed lines) of WT (blue) or PRF (red) parasites (*n* = 15 each). Parasitemia was monitored daily by microscopic examination of Giemsa stained blood-films. Differences between asexual blood-stage propagation were non-significant (Mantel-Cox test). **(B)** Kaplan-Meier analysis of time to development of signature symptoms of experimental cerebral malaria (ECM). ^***^P < 0.001 (Mantel-Cox test). **(C)** Quantification of parasite loads in the brain (square), BM (polygon) and liver (triangle) was done by qPCR. Organs were harvested 6 days after infection of C57BL/6 mice with 5,000 WT or PRF sporozoites. Animals were perfused via the left heart ventricle to remove non-sequestering infected red blood cells from the circulation of blood. Relative expression levels of *P. berghei* 18S rRNA were normalized to mouse *GAPDH*. Results represent mean values (±SD). ^*^P < 0.05 (Mann-Whitney test). **(D)** Visualization of integrity of blood-brain barrier in infected mice. Infected mice were intravenously injected with 2% Evans blue dye 8 days after infection by 5,000 WT sporozoites (upper left), PRF sporozoites (upper right), WT- iRBCs (bottom left), and PRF-iRBCs (bottom right). **(E)** Vascular leakage also occurs in the spinal cords of mice with ECM symptoms. Isolated spinal cords are indicated by arrows and show leakage of the dye in mice with signature ECM symptoms.

We next studied blood infection and intravenously injected 5,000 sporozoites or infected red blood cells (iRBCs) from WT or PRF parasites into naïve mice (Figure 2A). Quantification of parasitemia and growth dynamics revealed similar numbers of iRBCs. Strikingly, the majority of mice infected with PRF sporozoites (86%) did not develop ECM (Figure 2B). Of 22 mice that were infected with PRF sporozoites, only three mice developed signatures of ECM-like symptoms on day 7–8 after inoculation, while all other mice remained symptom-free as indicated by rapid murine coma and behavior scale (RMCBS) scores of 18 (29). In contrast, all mice infected with WT or PRF blood-stage parasites and all mice infected by WT sporozoites developed signature ECM symptoms (RMCBS scores of 0–4). PRF sporozoite-infected mice that survived the critical period of ECM onset (days 7–10 after inoculation) progressed to high parasitemia, and as a consequence, anemia several weeks later (Supplementary Figure 2).

To obtain more information about the development of blood-stage PRF parasites *in vivo*, the accumulation of parasites was analyzed in the brain, bone marrow (BM), and liver in perfused animals on day 6 p.i. with 5,000 sporozoites (Figure 2C), where no apparent difference between PRF and WT parasite burden in the three organs was observed. Altogether, these data demonstrate that PRF blood-stage parasites are fully virulent. Therefore, we infer from the markedly reduced incidence of ECM in mice infected with PRF sporozoites that the initial, pre-erythrocytic stage of a malarial infection profoundly impacts on the development of ECM.

### Reduced Inflammation of Cerebral Vessels in PRF Sporozoite-Infected Mice

**Figure 3:**
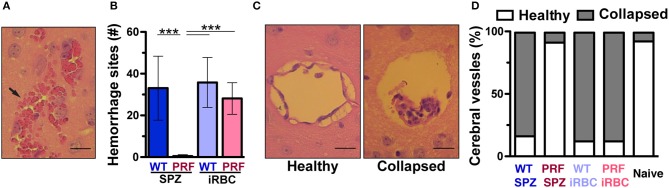
Cerebral vessels from mice infected by PRF sporozoites maintain a healthy status. **(A)** Hematoxilin and eosin (H&E) stain of a brain tissue section from an animal displaying signature ECM symptoms. An arrow indicates RBCs that leaked into the cerebral parenchyma. Scale bar, 10 μm. **(B)** Quantification of hemorrhage sites based on H&E stains of horizontal cross-sections of the brain (*n* = 10 each). Results represent mean values (±SD) from at least two independent experiments. ^***^P < 0.001 (Mann-Whitney test). **(C)** Visualization of brain vessel integrity. Shown are H&E stains of representative brain histological cross-sections. Comparison of a healthy vessel (left) from a mouse infected with PRF sporozoites and a collapsed vessel (right) from a mouse infected with WT sporozoites. The latter vessel also contains accumulated leukocytes. Scale bar, 10 μm. **(D)** Morphological scoring of cerebral vessels based on histological sections. Quantification of mice infected with 5,000 WT sporozoites, PRF sporozoites, WT iRBCs or PRF iRBCs (*n* = 10 animals for infected groups, *n* = 3 mice for naïve group; >20 cerebral vessels were quantified for each animal).

We compared the integrity of the BBB in infected mice as visualized by the coloration of the brain after intravenous infection of 2% Evans Blue dye, an indicator of BBB leakage (Figure 2D). Mice infected with WT sporozoites, WT iRBCs, and PRF iRBCs all showed strong diffusion of Evans Blue in the brain on day 7 or 8 p.i., while naïve mice and mice infected with PRF sporozoites remained unstained. We also observed vascular pathology in the spinal cord, which correlated with ECM (Figure 2E), a previously unreported finding. We propose that the progression of ECM extends to the spinal cord, which may be due to the disruption of the choroid plexus that acts as a blood-cerebrospinal fluid barrier (30). Furthermore, sites of hemorrhage i.e., areas where RBCs had leaked into the cerebral tissue were quantified by H&E stain of histological sections (Figure 3A). While hemorrhage sites were frequent in brain tissue derived from infected mice with ECM signatures, they were rarely detected in PRF sporozoite-infected mice (Figure 3B).

Additional key histopathological alterations associated with ECM are cerebral vessel plugging by RBCs/iRBCs, leukocytes and platelets (10, 31). Visualization of vessel plugging based on H&E stain showed vessels that were collapsed within an enlarged perivascular space in WT parasite-infected mice (Figure 3C). In contrast, cerebral vessels from naïve mice or mice infected with PRF sporozoites displayed healthy perivascular spaces, with the endothelium attached to surrounding tissues and almost no leukocyte infiltration (Figure 3C). From these sections, similar proportions (~90%) of healthy vessels were scored for naïve and PRF sporozoite-infected animals (Figure 3D). As expected from the associated pathology, mice infected with WT sporozoites, WT iRBCs, and PRF iRBCs displayed vessel plugging in the vast majority (~80%) of their cerebral vessels (Figure 3D). In conclusion, mice infected with PRF sporozoites displayed little morphological alteration of cerebral vessels despite similar blood-stage parasite abundance.

### Accumulation of Parasite Antigen-Specific CD8^+^ T Cells in the Brain and Spleen Is a Signature of Infection, but Not of ECM Development

**Figure 4:**
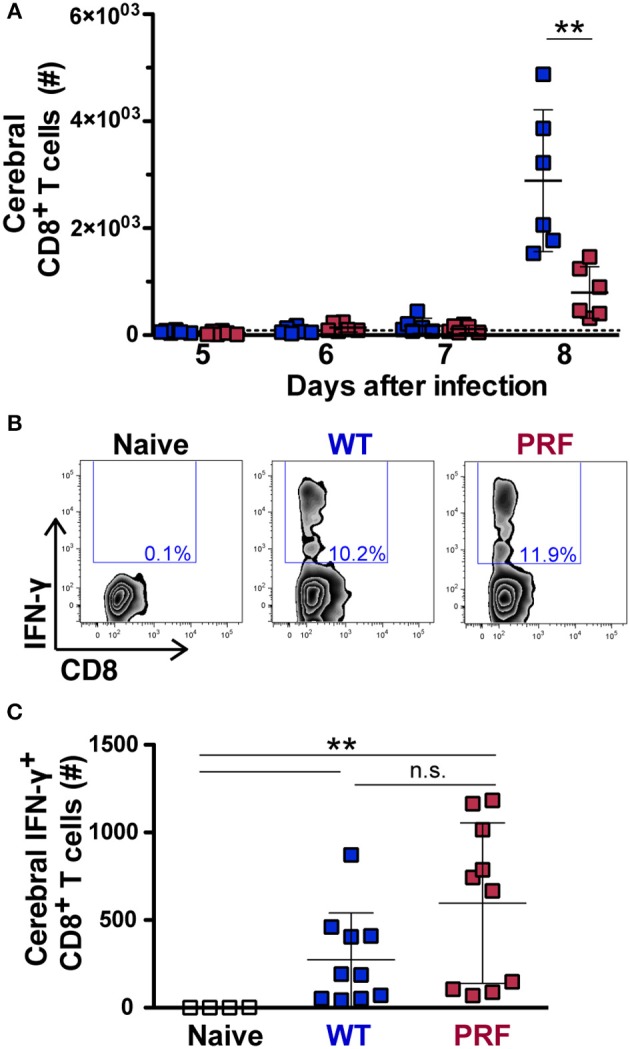
Quantification of total and antigen-experienced CD8^+^ T cells in the brains of infected mice. **(A)** Time course of total cerebral lymphocyte counts during the late stages of blood infection. Mice were infected with WT (blue) or PRF (red) sporozoites by intravenous injection (*n* = 6 each), and cerebral lymphocytes were isolated and analyzed at indicated time points. Shown are total CD8^+^ T cell numbers. The dotted line represents the mean cerebral CD8^+^ T cell number in all naïve animals (*n* = 16). The scatter dot plots represent mean values (±*SD*). ^**^P < 0.01 (Mann-Whitney test). **(B)** Gating strategy for detection of intracellular IFN-γ expression in CD3^+^CD8^+^ cells after re-stimulation with *Pb*GAP50_40−48_ peptide. Shown are representative plots for naïve animals, as well as mice infected with WT or PRF sporozoites. **(C)** Quantification of cerebral CD8^+^ T cells expressing intracellular IFN-γ after re-stimulation with *Pb*GAP50_40−48_ peptide. Shown are numbers of this population in the brain. The scatter dot plots represent mean values (±SD) from samples isolated on day 8 from three independent experiments (*n* = 10 each for infected mice; *n* = 4 for naïve mice). IFN-γ-secreting antigen-specific CD8^+^ T cells number from WT vs. PRF infected animals is non-significant. ^**^P < 0.01 (Mann-Whitney test).

The identification of parasite antigen-specific CD8^+^ T cells recognizing an epitope of *Plasmodium* glideosome-associated protein 50 (GAP50) allows for their quantification in the brain during infection (22). We first analyzed the expansion of splenic CD8^+^ T cells from day 5–8 after WT and PRF sporozoite-induced infections by flow cytometry, where the CD8^+^ T cell number was not significantly different between the two infections (Supplementary Figure 3A). The accumulation of CD8^+^ T cell number was significantly increased in infected animals compared to naïve mice on day 8 p.i., where the increase was more prominent for the ECM-developing group (Figure 4A). We next investigated the proportion of antigen-specific CD8^+^ T cells by measuring intracellular IFN-γ in isolated cerebral lymphocytes after re-stimulation with the GAP50_40−48_ peptide (Figure 4B). The IFN-γ-secreting antigen-specific CD8^+^ T cells were not significantly different in the spleen (Supplementary Figures 3B,C) on day 8 after sporozoite infection. Furthermore, cerebral analysis revealed that the numbers, percentages and even the mean fluorescence intensity of IFN-γ-secreting antigen-specific CD8^+^ T cells were not significantly different after both infections in brain (Figure 4C and Supplementary Figure 4). Other lymphocyte populations including CD4^+^ T, NKT, and NK cells accumulated in the brains on day 8 p.i., as previously published (31), although this occurred in lower amounts in mice infected with PRF sporozoites compared to mice that received the WT parasite (Supplementary Figure 5), as observed for total CD8^+^ T cells (Figure 4A).

Taken together, our data show that infections with PRF sporozoites elicit high frequency of IFN-γ-secreting antigen-specific CD8^+^ T cells, yet trigger a lower accumulation of total immune cells in the brain, as compared to WT sporozoites. This finding is reminiscent of what was observed in mice infected with different strains of *P. berghei* (20, 22). Our data likely reflect diverse degrees of re-activation of parasite-specific CD8^+^ T cells locally in the brain, so that there is reduced endothelial cell activation upon PRF sporozoite infections.

### Parasite Infection Leads to Similar Cerebral Myeloid Populations Regardless of Disease Outcome

**Figure 5:**
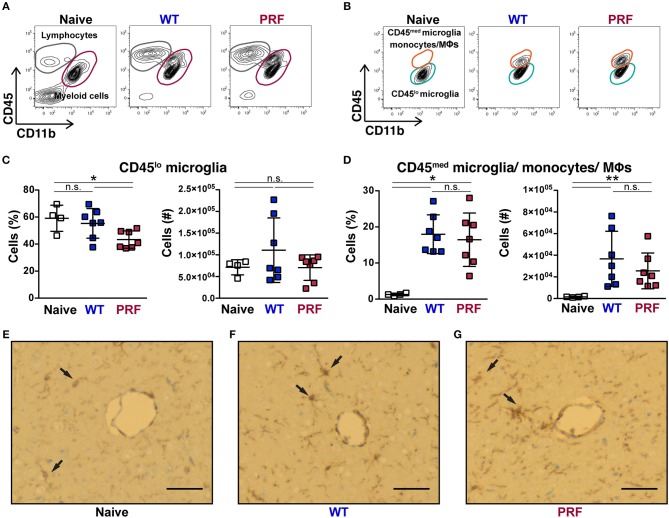
Quantification of myeloid cells after infection. **(A)** Representative contour plots from day 8 after infection showing CD45 vs. CD11b expression on live cells obtained from naïve mice (left), and mice infected with WT (center) and PRF (right) sporozoites. Indicated are gated populations of lymphocytes, CD45^hi^CD11b^−^ cells (gray circles) and myeloid cells, CD45^med/lo^CD11b^+^ cells (red circles). **(B)** CD45 vs. CD11b expression on the Ly6G^−^ myeloid population. CD45^lo^CD11b^+^ cells (orange circles) represent microglia and CD45^med^CD11b^+^ cells (green circles) activated microglia, monocytes and macrophages, respectively. **(C)** Quantification of CD45^lo^ microglia. Shown are percentage (left) and absolute numbers (right) of CD45^lo^Ly6G^−^CD11b^+^ microglia. **(D)** Quantification of CD45^med^Ly6G^−^CD11b^+^ cells, which correspond to CD45^med^ microglia, monocytes and macrophages. Shown are percentage (left) and absolute numbers (right) of CD45^med^ microglia/monocytes/macrophages in the brain. The scatter dot plots in **(C,D)** represent mean values (±*SD*) from samples (*n* = 4–7) isolated 8 days after infection from two independent experiments. n.s, non-significant; ^*^P < 0.05; ^**^P < 0.01 (Mann-Whitney test). **(E–G)** Representative images from the IBA-1^+^ microglial cells of brain histological cross-sections. Cerebral vessels are indicated by arrows. The IBA-1 staining reaction was visualized with diaminobenzidine (DAB), highlighting some microglial cells with thin processes in naïve mice, while they are more prominent in WT sporozoite-infected mice and they also tend to cluster around vessels. This feature is also pronounced in PRF sporozoite-infected mice, dark brown. Scale bar, 50 μm.

Although the contribution of myeloid cells to ECM remains unclear, these cells represent suitable sensors of an infection locally. We, thus, examined the cerebral myeloid populations in our infection models. Cerebral myeloid populations were defined as CD45^med/lo^CD11b^+^ cells, which excluded lymphocytes (Figure 5A). We further excluded Ly6G^+^ neutrophils, which were not significantly different between the three groups (Supplementary Figure 6). Based on their CD45 expression, myeloid cell populations were separated into CD45^lo^ microglia and CD45^med^ microglia, monocytes or macrophages, which are difficult to distinguish further (Figure 5B). Although it has been reported recently that non-ECM animals display lower numbers and less activation of microglia (32), we found that the CD45^lo^ microglia number was not significantly different between naïve, WT sporozoite- and PRF sporozoite-infected animals (Figure 5C). There was a comparable increase in the number of CD45^med^ microglia, monocytes and macrophages in both groups of infections (Figure 5D). Moreover, the activation of microglia in infected mice was also apparent from the ionized calcium-binding adapter molecule 1 (IBA-1) immunostaining of brain cross-sections (Figures 5E–G). We detected high IBA-1 expression especially in proximity to the vessels, where microglia were exposed to parasite antigens, inflammatory cytokines, and chemokines. Thus, microglia activation was overall comparable after WT or PRF parasite infections. We also determined that during WT and PRF sporozoite infections most of the cells in this population were Ly6C^hi^ pro-inflammatory monocytes (Supplementary Figure 7A), and their accumulation in the brain was similar for both groups of infections (Supplementary Figure 7B). In summary, we found no difference in the phenotype of myeloid populations in the brains from WT- and PRF sporozoite infected-mice, which is in perfect agreement with our observation of similar parasite growth and abundance in the brain during both infections.

**Figure 6:**
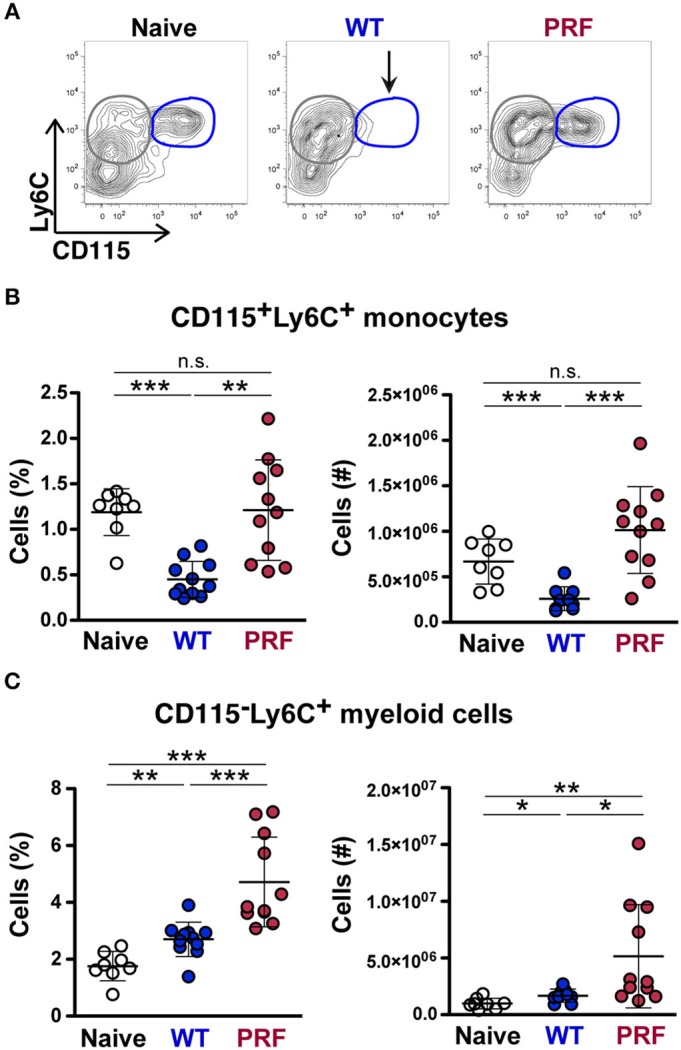
Reduced CD115 expressions on splenic Ly6C^+^CD11b^+^ monocytes in mice infected with WT sporozoites during the development of ECM. **(A)** Representative contour plots showing gating of Ly6C vs. CD115 expression on CD45^+^Ly6G^−^CD11b^+^ myeloid cells from naïve mice (left), and mice infected with WT (center) and PRF (right) sporozoites 8 days after infection. An arrow indicates the disappearance of CD115^+^Ly6C^+^ monocytes (blue circles) in spleens of mice infected with WT sporozoites. CD115^−^Ly6C^+^ monocytes are gated in gray circles. **(B)** Quantification of splenic CD115^+^Ly6C^+^ monocytes. Shown are percentage and numbers of CD115^+^Ly6C^+^ monocytes in the spleen. **(C)** Quantification of splenic CD115^−^Ly6C^+^ cells. Shown are percentage and numbers of CD115^−^Ly6C^+^ myeloid cells in the spleen. The scatter dot plots in **(B,C)** represent mean values (±SD) from samples (*n* = 8–11) isolated 8 days after infection from two independent experiments. n.s, non-significant; ^*^P < 0.05; ^**^P < 0.01; ^***^P < 0.001 (Mann-Whitney test).

Interestingly, while investigating Ly6C^+^ monocytes in the spleen of mice infected with WT or PRF sporozoites on day 8 after infection, we found that mice with onset of ECM displayed a striking loss of CD115^+^Ly6C^+^ monocytes in spleen, which was neither apparent in PRF sporozoite-infected nor in naïve mice (Figures 6A,B). Moreover, the decrease in the number of CD115^−^Ly6C^+^ myeloid cells in the spleen of mice infected with WT sporozoites was also pronounced in comparison to PRF sporozoite-infected mice (Figure 6C). CD115 can be used to distinguish monocytes from macrophages (33), and CD115 is a receptor for macrophage colony-stimulation factor (M-CSF) and IL-34 (34).

### PRF Parasites Induce Higher Systemic Cytokines Levels at the Time of Their Emergence From the Liver

**Figure 7:**
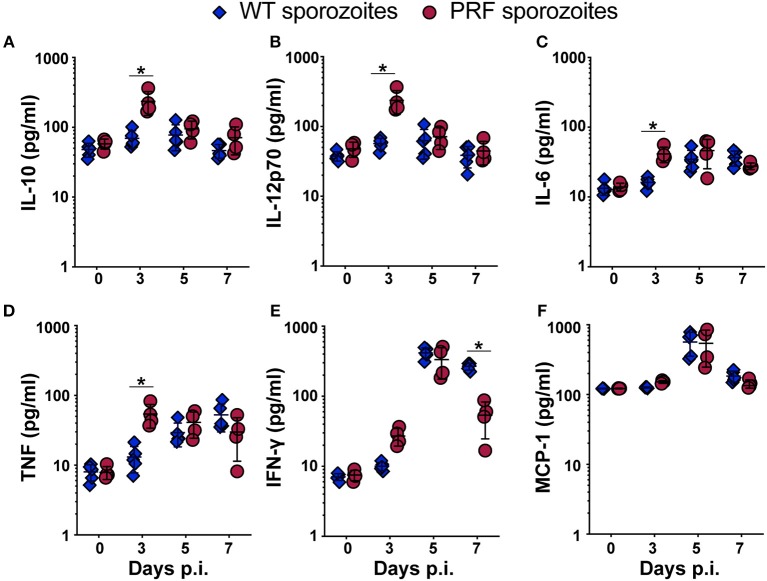
Serum cytokine levels are up-regulated 3 days after infection with PRF sporozoites, at the onset of blood infection. **(A–F)** Serum from peripheral blood was collected on days indicated after infection with 5,000 WT (blue triangles) or PRF (red circles) sporozoites. Systemic cytokines were captured using a Cytokine Bead Array and analyzed by flow cytometry. Day 0 results represent data from uninfected mice. Shown are the steady-state levels of the systemic cytokines **(A)** IL-10, **(B)** IL-12p70, **(C)** IL-6, **(D)** TNF, **(E)** IFN-γ, and **(F)** MCP-1. The results represent mean values (±*SD*) (*n* = 5 for WT, *n* = 4 each for PRF). ^*^P < 0.05 (Mann-Whitney test).

To address whether PRF sporozoites induced a different immune response during the liver phase in comparison to WT parasites we quantified the systemic cytokine levels in the blood on days 0, 3, 5, and 7 after sporozoite-induced infections (Figure 7). In PRF sporozoite infections, we observed an abrupt rise in the serum levels of the regulatory cytokine interleukin-10 (IL-10), which is known to be the key suppressive cytokine implicated in ECM prevention (35). Strikingly, IL-10 up-regulation was only observed on day 3 p.i. (Figure 7A), coinciding with the time point when transgenic blood-stage parasites emerged from the liver into the blood circulation (Figure 1A). We also observed an increase in IL-12p70, IL-6, and tumor necrosis factor (TNF) on day 3 after PRF sporozoite infections as compared to WT sporozoite infections (Figures 7B–D). Of note, an early production of cytokines, such as IL-10 and IL-12 can be protective rather than deleterious in ECM pathology (36, 37).

Mice infected with PRF sporozoites displayed lower systemic IFN-γ levels than mice infected with WT sporozoites at day 7 p.i., which is consistent with a lower re-activation of CD8^+^ T cells in PRF parasite-infected mice at the time when mice infected with WT parasites develop ECM (Figure 7E). These changes were specific since the levels of other soluble factors, such as monocyte chemo attractant protein-1 (MCP-1), were similar in both infections (Figure 7F).

### PRF Pre-erythrocytic Parasites Mount Reduced Hepatic Immune Responses

**Figure 8:**
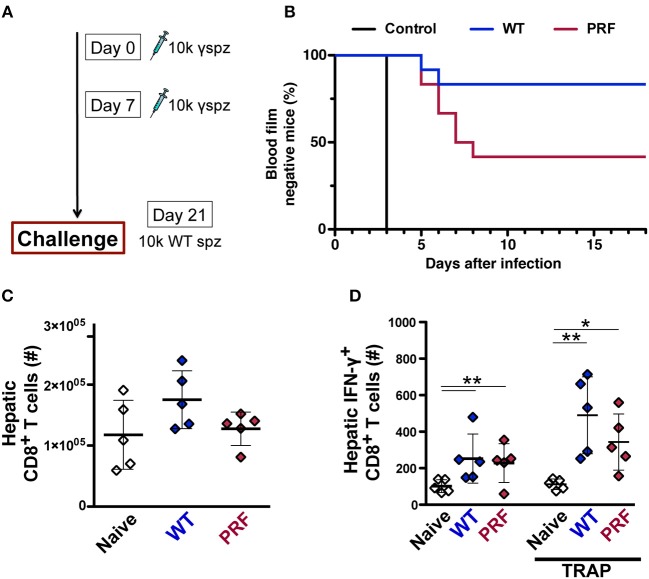
PRF sporozoites do not induce more liver-stage antigen specific CD8^+^ T cells compared to WT sporozoites upon immunization. **(A)** Immunization and challenge protocol. C57BL/6 mice were immunized twice at weekly intervals with 10,000 irradiated hemocoel sporozoites (10 k γ-spz). Animals were challenged with 10,000 WT salivary gland sporozoites (10 k spz) 14 days after the last immunization. **(B)** Kaplan-Meier analysis of time to blood infection. Naïve mice (*n* = 9), WT and PRF sporozoite-immunized mice (*n* = 12 each). Blood parasitemia was determined by daily microscopic examination of Giemsa-stained blood films. The statistics for WT vs. PRF sporozoite-immunized mice were non-significant for Log-rank (Mantel-Cox) test. **(C)** Enumeration of total liver CD8^+^ T cell numbers after 14 days from the last immunization. Livers were isolated for flow cytometric analysis from naïve and WT and PRF sporozoite-immunized mice (*n* = 5). The results represent mean values (±*SD*). **(D)** Quantification of IFN-γ-secreting antigen-specific liver CD8^+^ T after 14 days from the last immunization. Hepatic leukocytes were stained for intracellular IFN-γ production without (left) or after re-stimulation with *Pb*TRAP_130−138_ peptide (right). The results represent mean values (±*SD*). ^*^P < 0.05; ^**^P < 0.01 (Mann-Whitney test).

To explore whether the pre-erythrocytic phase of infection with transgenic parasite modifies the subsequent immune response, we used irradiation-arrested sporozoites that are capable of invading the host hepatocytes, yet maturation is halted during early liver-stage development. This approach is typically used to study the sterile immunization by the whole-sporozoite vaccination strategies, but it also allows the study of hepatic immune responses elicited by the early pre-erythrocytic phase of the infection. Two doses of weekly intravenous inoculations with 10,000 irradiated WT or PRF hemocoel sporozoites were followed by a challenge infection by 10,000 WT salivary gland sporozoites 14 days after the last immunization (Figure 8A). Sterile protection was determined by daily monitoring of parasitemia after the challenge infection. As expected, naïve mice became blood-stage positive on day 3 after infection. Only 2 out of 12 mice immunized with irradiated WT hemocoel sporozoites became infected and the remaining 10 mice were free of blood-stage infection throughout the observation period of 3 weeks. In marked contrast, 7 out of 12 PRF hemocoel sporozoite-immunized mice developed parasitemia starting 5 days after the challenge infection (Figure 8B). In conclusion, these data show that immunization with PRF hemocoel sporozoites induces weaker immunity, despite the enhanced cytokine production and sporozoite activity.

We further studied the expansion of hepatic CD8^+^ T cells by pre-erythrocytic parasites using irradiated sporozoites. We found no differences in the total CD8^+^ T cells after two doses of weekly intravenous inoculation with 10,000 irradiated WT or PRF hemocoel sporozoites (Figure 8C). However, there was a marginally reduced number of IFN-γ-secreting *Pb*TRAP_130−138_-specific CD8^+^ T cells expansion in PRF sporozoite-immunized mice as compared to WT sporozoite-immunized mice (Figure 8D), in good agreement with the inferior ability of irradiated PRF sporozoites to mount sterilizing immune responses (Figure 8B). This finding suggests that the enhanced early production of cytokines by PRF sporozoites is linked to dampening of cell-based protective immune responses in the liver, similar to the dampening of pathogenic immune responses associated with ECM disease onset.

## Discussion

The utilization of a transgenic parasite line, which displays enhanced hepatocyte transmigration and invasion, allowed us to identify a novel checkpoint in ECM disease onset. We propose that this checkpoint is defined by signatures of cellular immunology and can be modulated to the benefit of *Plasmodium*-infected hosts. Only few studies addressed how the pre-erythrocytic phase of the infection impacted on ECM, either employing chemical attenuated- (38) or knock-out *P. berghei* ANKA parasites (32, 39, 40), which are no longer virulent. Thus, the prevention of cerebral pathology in these infections can be largely explained by considerable delays in the time to blood infection or by strongly reduced parasite virulence and lower infection rates, which do not induce the threshold of pro-inflammatory responses required for ECM. In marked contrast, PRF sporozoites displayed no apparent attenuation, yet failed to induce cerebral pathology in sporozoite-induced infections, while emerging normally from the liver and being fully virulent when the infection was induced by blood transfusion of iRBCs.

Irrespective of the parasite strains used and the outcome of brain pathology, accumulation of IFN-γ-secreting antigen-specific CD8^+^ T cell in the brain was readily altered, which has been similarly observed in previous studies using ECM inducing ANKA and non-ECM inducing NK65 *P. berghei* strains (20, 22). In fact, it has been demonstrated that vascular pathology is triggered when these parasite antigen-specific CD8^+^ T cells interacted with IFN-γ activated cerebrovascular endothelial cells that cross-present malaria antigens (21–23, 40). PRF sporozoite infections show healthy cerebral microvessels, but further work will be required to confirm the molecular mechanism of protection from the vascular destruction by examining factors such as cytotoxicity of CD8^+^ T cells, cerebral chemokine levels and other increased populations like CD4^+^ T, NKT, and NK cells.

We did not observe any differences in cerebral myeloid populations in all infected animals, irrespective of disease propagation, suggesting that myeloid cells respond to the similar amount of blood-stage parasites in both infections and reflect peripheral sensing of inflammation. An interesting observation in this study is the lack of CD115 marker on Ly6C^+^ monocytes in the spleen of mice with ECM symptoms. M-CSF signaling via CD115 is important for the late stage of monocyte and macrophage lineage development (41), where in a similar manner to fatal ECM pathology, a lethal *Listeria monocytogenes* infection has also been reported to induce a down-regulation of CD115 expression on monocytes (42). We could not directly test the possibility of infiltration of CD115^+^Ly6C^+^ monocytes to the brain due to cleavage of CD115 after the collagenase treatment during the cerebral leukocyte isolation (43). Further investigation is warranted to characterize and identify the different subsets of Ly6C^+^ monocytes during ECM and other pathological conditions of *Plasmodium* infections.

A failure to establish an appropriate balance between pro- and anti-inflammatory immune responses is believed to be central to the development of cerebral pathology (44). Profilin from the distantly related coccidian parasite, *Toxoplasma gondii*, has been shown to be a key immunomodulatory protein that is sensed by the murine-specific Toll-like receptor 11 (TLR11), leading to activation of the myeloid differentiation primary response protein 88 (MyD88) pathway in murine dendritic cells (45). Activation of dendritic cells by *T. gondii* profilin induces IL-12 followed by IFN-γ production from NK cells and neutrophils, which predetermines host resistance to *Toxoplasma* infection (46). Systemic up-regulation of IL-12 on day 3 p.i., together with IL-10, TNF, and IL-6, reflects the early checkpoint and is a major contributor to the prospective lack of ECM pathology after PRF sporozoite infection. Although induction of TLR11 was not observed by recombinant *Plasmodium* profilin (45), we cannot formally exclude that artificially elevated levels of *Plasmodium* profilin also contribute to the observed phenotype, in addition to an enhancement of parasite transmigration and liver stage development. While we suggest that the elevated early cytokine responses signify a host response to enhanced cellular transmigration, penetration and invasion of sinusoid cells and hepatocytes by transgenic sporozoites, we cannot formally exclude that higher expression of the otherwise inert *P. berghei* profilin or other proteins driven by the genetic modification in sporozoites might also contribute to the immune modulatory property of PRF parasites. Such a role would, however, be specific to sporozoites, since similar expression in merozoites (25) does apparently not trigger such a response, as seen by full virulence of infections with PRF iRBCs.

Interestingly, despite the enhanced sporozoite activity and elevated early cytokine responses, the expansion of antigen-specific CD8^+^ cells in the liver and sterilizing immunity were inferior in immunizations with irradiated PRF sporozoites. At this stage, we cannot determine the level of antigen exposure by PRF sporozoites; however there appears to be a dampening effect to the subsequent T cell immunity elicited specifically by PRF pre-erythrocytic parasites. Careful examination of detailed morphology during the pre-erythrocytic stages, such as formation of vacuoles and tubular protrusions will be needed to unravel the protective scenario of a liver-stage infection that can ultimately prevent the ECM development.

Further studies on how transgenic sporozoites and liver-stage parasites develop and interact with the host immune cells are critical to delineate the involvement of hepatic immunity to affect the blood-stage outcome. Besides the CD8^+^ T cells that we have examined in this study, recent findings by others on γδ T cells in *Plasmodium* infection may shed light on the underlying immune mechanism that determines the prevention of ECM during PRF sporozoite infection. Absence of IFN-γ-producing γδ T cells during the liver-stage infection resulted in a less pro-inflammatory microenvironment that prevented mice from ECM development, demonstrating the role of hepatic immunity on cerebral pathology (47). In another study using *P. chabaudi* infection induced IFN-γ-secreting γδ T cells during the early infection, while γδ T cells shifted to M-CSF secretion toward later time points in infection (48).

In conclusion, our study demonstrates the critical importance of the pre-erythrocytic phase of infection, which is clinically silent. Additional work is warranted to uncover the molecular and cellular mechanisms of the entire cascade that commences with the first sporozoite-host interaction and ultimately leads to the detrimental brain pathology. Whether modulation of the early pre-erythrocytic immune response could also dampen cerebral malaria in *P. falciparum*-infected patients remains to be determined for the development of novel adjunct therapies and early diagnoses that are urgently needed to alleviate the fatal outcomes of cerebral malaria in patients.

## Materials and Methods

### *Plasmodium berghei* Parasites

*Anopheles stephensi* mosquitoes were raised at 20°C in 75% humidity under a 14-h light/10-h dark cycle. NMRI mice were infected by intraperitoneal injection of wild type *P. berghei* (strain ANKA) (49) or the transgenic parasite line expressing profilin under the control of the *AMA-1* promoter (PRF) (25). iRBCs were collected from a drop of blood obtained from the tail vein. Sporozoites were isolated from the hemocoel of infected mosquitoes from day 17–22 after an infectious blood meal (28). For blood-stage infection, 5,000 iRBCs or freshly isolated sporozoites were injected intravenously into C57BL/6 mice. To exclude accumulation of unrecognized mutations, stocks of PRF iRBC were generated from sporozoite-induced infections, which were confirmed to not display ECM signatures. Microscopic examination of daily Giemsa-stained blood smears was conducted to determine parasitemia. Approximately, 20,000 RBCs were screened to determine the prepatent period.

### Histopathological Scoring of Brain Sections

Mice were intravenously injected with 200 μl of 2% Evan's blue dye (Sigma-Aldrich) prepared in PBS. After 5–10 min, mice were sacrificed and brain and spinal cord were carefully dissected, rinsed quickly in PBS, and photographed. Histopathological scoring of brain sections was prepared from perfused mice. Organs were fixed in 2 ml of 4% formaldehyde for 24 h and embedded in paraffin blocks. H&E staining was done on 4 μm horizontal sections. Microglia were stained using anti-IBA1 antibody (Wako Chemicals GmbH). Hemorrhage sites and the status of cerebral vessels were scored microscopically.

### Leukocyte Isolation

Leukocytes from spleen, BM and brain were isolated on days indicated after perfusion with 1xPBS. Brains were collected in PBS/1% BSA/1 mM EDTA on ice, transferred, and incubated in digestion solution containing 0.02% collagenase type I (Sigma-Aldrich) and 0.002% DNase I (Sigma-Aldrich) in RPMI medium for 5 min at 37°C. Samples were incised into tiny sections then incubated for 10 min at 37°C. Cells were filtered into 15 ml falcon and centrifuged for 5 min, 1,400 rpm at 4°C. Pellets were resuspended in 5 ml 30% Percoll (Sigma-Aldrich) and centrifuged for 20 min, 2,000 rpm without break at room temperature. Fatty layer on top and the supernatant were aspirated. The cell pellets were resuspended in complete RPMI as described previously (50). Marrows of tibia were extracted by flushing the bone. Marrow and spleen were grinded between two glass slides, and then treated by Gey's solution (0.155M NH_4_Cl and 0.01M KHCO_3_) to lyse the RBCs. Cells were stained with DAPI (Thermo Fisher Scientific) to exclude the dead cells and quantified using MACS Quant Analyzer 10. Isolated cerebral cells were stained for anti-mouse CD45.2-APC antibody (clone 104; eBioscience) to quantify the leukocyte population.

### Leukocyte Surface Staining

Cells collected from brain, spleen, or BM were filtered through 50-μm cell strainer (Partec) and blocked with antibodies against Fc-receptors (CD 16/32, clone 2.4G2; in house). Cells were stained with following anti-mouse antibodies for the time course experiment: CD3e-PeCy7 (clone 145-2c11; eBioscience), TCRβ-APC (clone H57-597; eBioscience), CD4-FITC (clone RM4-5; eBioscience), CD8-BV570™ (clone 53-6.7; Biolegend), CD49b (clone DX5; in house), and Propidium Iodine (Thermo Fisher Scientific) to test cell viability. The myeloid cell population was stained with the following antibodies: CD45.2-APC, CD11b-Pacific Blue (clone M1/70; in house), Ly6G-FITC (clone 1A8-Ly6g; eBioscience), Ly6C-Biotin (clone AL-21, BD Biosciences), CD115-PE (clone AF598, eBioscience), streptavidin-PerCP (BD Biosciences), and Pacific Orange-N-Hydroxysuccinimide (NHS) (in house) as viability dye. Stained live cells were acquired (stopping gate of 100,000 live cells) using a MACS Quant Analyzer 10 and analyzed by FlowJo software.

### Re-stimulation and Intracellular Staining of Lymphocytes

3 × 10^6^ isolated leukocytes were distributed in 96-well plates and resuspended in 100 μl of complete RPMI medium. The immunogenic peptides *Pb*GAP50_40−48_ (SQLLNAKYL-NH2; peptides&elephants, Hennigsdorf, Germany) (22) and *Pb*TRAP_130−138_ (SALLNVDNL-NH2; peptides&elephants, Hennigsdorf, Germany) (51) were used. 50 μl peptide diluted in RPMI (10 g/ml) was incubated for 2 h at 37°C, 5% CO_2_. Thereafter, 50 μl of Brefeldin A (1:1,000; eBioscience) was added to block secretion of cytokines, and cells were incubated for additional 4 h. After centrifugation cells were stained for surface markers with CD3e-PeCy7, CD4-BV421™ (clone RM4-5; Biolegend) and CD8-BV570™ antibodies, followed by staining with Pacific Orange-NHS for cell viability. Cells were fixed with 4% paraformaldehyde for 15 min at room temperature, and then permeabilized with Perm/Wash™ buffer (BD Biosciences). Intracellular IFN-γ was stained with Interferon gamma-APC antibody (clone XMG 1.2; eBioscience). Cells were washed in Perm/Wash™ buffer, then resuspended in PBS/1% BSA for acquisition (stopping gate of 20,000 CD8^+^ T cells) with MACS Quant Analyzer 10 and analysis by FlowJo software (51).

### Plasma Cytokine Measurements

C57BL/6 mice were infected intravenously with 5,000 sporozoites. Blood was collected from the tail in a heparinized micro-hematocrit capillary on days indicated. Samples were centrifuged at 13,000 rpm for 3 min and plasma collected and stored at −80°C. Plasma cytokines were assayed by a cytometric bead array (mouse inflammation kit; BD Biosciences) as described previously (37). Analysis was performed using a Fortessa cell analyzer (BD Biosciences) and FlowJo software.

### Liver-Stage Development

For the sporozoite cell traversal assay, 24-well plates were seeded with 300,000 human hepatoma cells (Huh7) per well and inoculated with 35,000 sporozoites in 300 μl of DMEM complete medium with 0.5 μg/ μl of FITC-dextran (Thermo Fisher Scientific) (52). After centrifugation for 5 min at 3,000 rpm, the plates were incubated for 30 min or 1 h at 37°C with 5% CO_2_. Trypsin-treated cells were resuspended in PBS, filtered, and immediately acquired by MACS Quant Analyzer 10 (Miltenyi Biotec) and analyzed by FlowJo software (Tree Star) to quantify dextran-positive, traversed cells.

To monitor successful parasite development, Huh7 cells were infected with 6,000 sporozoites isolated in DMEM. For settlement, the wells were centrifuged for 5 min at 3,000 rpm and incubated for 2 h at 37°C with 5% CO_2_. To stop cell invasion, the cells were washed three times with DMEM to remove extracellular sporozoites. Thereafter, cells were incubated for 24 or 48 h to permit development of liver-stage parasites. Cells were fixed with 4% paraformaldehyde for 10 min, followed by immunofluorescent assay with Hoechst 33342 (Thermo Fisher Scientific) and anti-*P. berghei* HSP70 antibody (53).

### Quantitative RT-PCR

Five thousand freshly dissected sporozoites were injected intravenously into C57BL/6 mice. Livers were isolated after 42 h of infection. For organs collected 6 days post-infection, animals were first perfused with 50 ml of PBS via the left heart ventricle to remove iRBCs in the periphery. Organs were rinsed and then homogenized in Trizol reagent (Thermo Fisher Scientific). Total RNA was isolated, and cDNA was synthesized (RETROscript, Thermo Fisher Scientific). qRT-PCR was performed with Power SYBR Green PCR master mix (Thermo Fisher Scientific) as described previously (54, 55) with the ABI 7500 sequence detection system (Thermo Fisher Scientific). Gene-specific primers for *P. berghei* 18S rRNA (gi:160641 [forward, 5′−*AAGCATTAAATAAAGCGAATACATCCTTAC*−3′; *reverse*, 5′]) and mouse *GAPDH* (gi:281199965 [forward, 5′−*TGAGGCCGGTGCTGAGTATGTCG*−3′; *reverse*, 5′−*CCACAGTCTTCTGGGTGGCAGTG*−3′]) were utilized for the amplification. The relative transcript abundance was determined using the 2^−ΔΔCt^ method.

### Immunization With Irradiated Sporozoites

Freshly dissected sporozoites were irradiated with 12,000 cGy. A total of 10,000 irradiated sporozoites were intravenously injected per immunization. Challenge experiments were carried out with 10,000 wild-type salivary gland sporozoites. Immunized animals were monitored for the presence of blood-stage parasites from day 3 onward until day 14 after challenge by daily microscopic examination of Giemsa-stained blood films. Sterile protection was defined as the complete absence of blood-stage parasites.

### Statistical Analysis

Statistics were conducted using GraphPad Prism 5 (GraphPad Software). Statistical significance was calculated using a Mann-Whitney test (non-parametric test). A *P* < 0.05 was considered significant. Survival curves were compared by using the log rank (Mantel-Cox) test. Kruskal-Wallis test was performed to compare the significance of dependent data.

## Data Availability Statement

All datasets generated for this study are included in the article/Supplementary Material.

## Ethics Statement

All animal work was conducted in accordance with the German Tierschutzgesetz in der Fassung von 18. Mai 2006 (BGB1. I S. 1207), which implements the Directive 86/609/EEC from the European Union and the European Convention for the protection of vertebrate animals used for experimental and other scientific purposes. The protocol was approved by the ethics committee of the Max Planck Institute for Infection Biology and the Berlin state authorities (Landesamt für Gesundheit und Soziales (LAGeSo permit number G0469/09). Female C57BL/6 and NMRI mice were ordered from Charles River Laboratories. Mice were diagnosed with onset of ECM if they showed behavioral and functional abnormalities, such as ataxia, paralysis, or convulsions (12, 29). Mice were sacrificed immediately after a diagnosis of ECM.

## Author Contributions

YS, SF, and KM planned the work. YS and SR performed experiments. YS analyzed the data. YS, SR, and WS contributed to data analysis. YS wrote the manuscript with input from WS, SF, and KM. The final manuscript was edited and approved by all authors.

### Conflict of Interest

The authors declare that the research was conducted in the absence of any commercial or financial relationships that could be construed as a potential conflict of interest.
