# Supplementary material for: The Liver-Stage Plasmodium Infection Is a Critical Checkpoint for Development of Experimental Cerebral Malaria
Source: Front Immunol. 2019 Nov 1;10:2554. doi: 10.3389/fimmu.2019.02554 (PMC6837997; doi:10.3389/fimmu.2019.02554)
Supplement: Supplementary file 1 [file Data_Sheet_1.pdf]

Supplementary Material

The Liver-stage *Plasmodium* Infection Is a Critical Checkpoint for Development of Experimental Cerebral Malaria

Yuko Sato, Stefanie Ries, Werner Stenzel, Simon Fillatreau, Kai Matuschewski

Content:

- **Supplementary Figures S1 –S7**

Sato *et al.*, Supplementary Figure S1

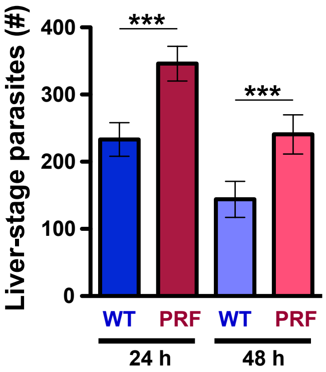

Supplementary Figure 1: *In vitro* development of liver-stage parasites.

Hepatoma cells were incubated with sporozoites for 24 and 48 h before fixation and stained with an anti-*Pb*HSP70 antibody. Results represent mean values (± SD) of at least three independent experiments with duplicate or triplicate samples. ***, P<0.001 (Mann-Whitney test).

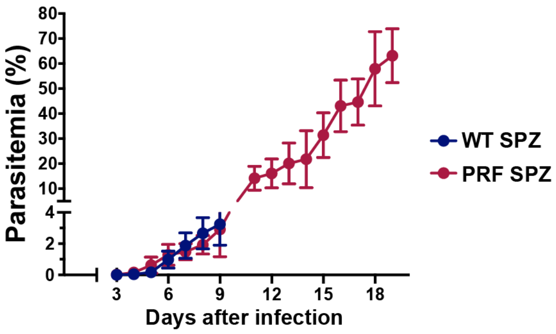

Supplementary Figure 2. PRF hemocoel sporozoite infection leads to hyper-parasitemia. Parasitemia was followed for a longer period of time for a proportion of PRF hemocoel sporozoite infected animals (*n*=22 each). Mice were sacrificed when they presented symptoms of ECM (WT sporozoite-infected) or severe anemia (PRF sporozoite-infected).

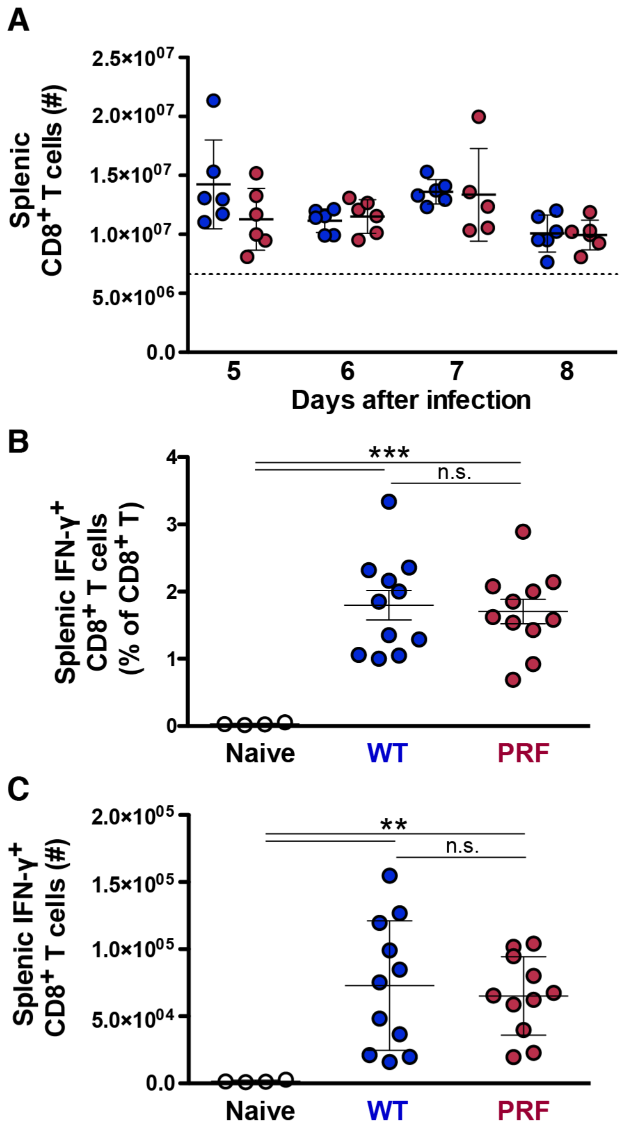

Supplementary Figure 3. Quantification of total and antigen-experienced CD8$^+$ T cells in the spleens of infected mice.

(A) Time course of total splenic CD8$^+$ T cells. Mice were infected with WT (blue) or PRF (red) sporozoites by intravenous injection (*n*=6 each), and splenocytes were isolated and analyzed at indicated time points. The dotted line represents the average total CD8$^+$ T cells from naïve animals. Results represent mean values (± SD) (*n*=6 each). Differences were non-significant (Mann-Whitney test).

(B-C) Quantification of splenic CD8$^+$ T cells expressing intracellular IFN-γ after re-stimulation with *Pb*GAP50$_{40\text{-}48}$ peptide. Shown are percentages out of splenic CD8$^+$ T cells (**B**) and numbers (**C**) of this population in the spleen. Splenic leukocytes were stained for intracellular IFN-γ production with *Pb*GAP50$_{40\text{-}48}$ peptide. Results represent mean values (± SD) (*n*=10 each for infected mice; *n*=4 for naïve mice). Samples were isolated 8 days after infection. Differences in IFN-γ-secreting antigen-specific CD8$^+$ T cell numbers from WT *versus* PRF infected animals are non-significant. **, P<0.01; ***, P<0.001(Mann-Whitney test).

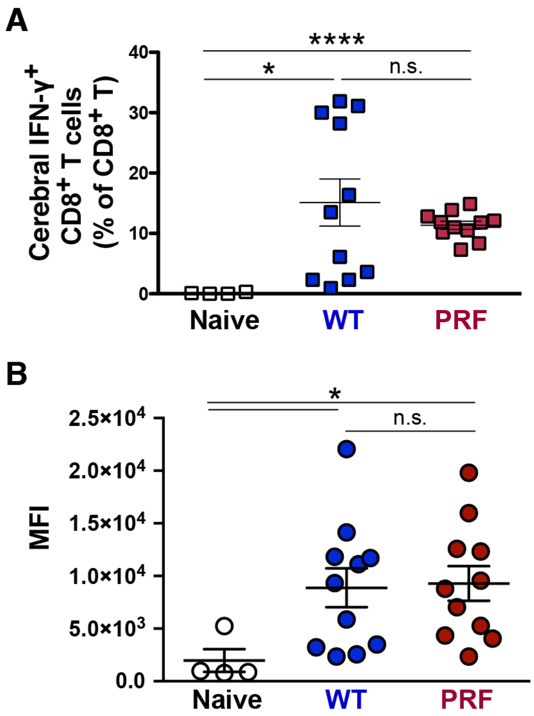

Supplementary Figure 4. Quantification of total and antigen-experienced $CD8^+$ T cells in the brain of infected mice.

Quantification of cerebral $CD8^+$ T cells expressing intracellular IFN-γ after re-stimulation with *Pb*$GAP50_{40-48}$ peptide. The scatter dot plots represent mean values (± SD) from samples isolated on day 8 from three independent experiments (*n*=10 each for infected mice; *n*=4 for naïve mice).

(A) Shown are percentages out of cerebral $CD8^+$ T cells of this population in the brain. Total percentage of IFN-γ-secreting antigen-specific $CD8^+$ T cells from WT versus PRF infected animals is non-significant. *, $P<0.05$; ****, $P<0.0001$ (Mann-Whitney test).

(B) Quantification of mean fluorescence intensity (MFI) of IFN-γ secreted from antigen-specific $CD8^+$ T cells in the brain by flow cytometry. n.s., non-significant; *, $P<0.05$ (Mann-Whitney test).

Sato *et al.*, Supplementary Figure S5

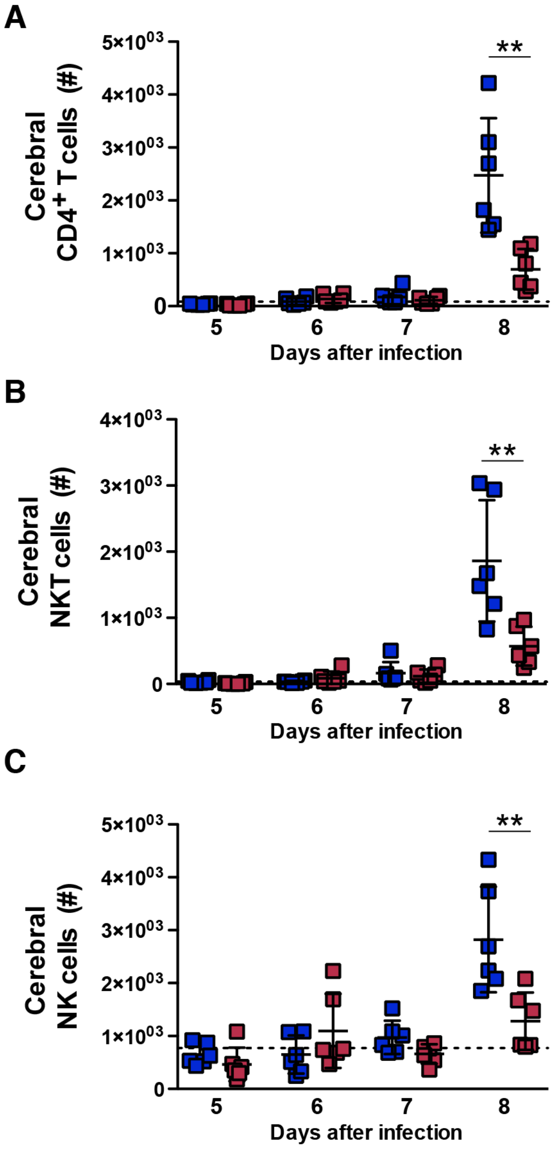

Supplementary Figure 5. Quantification of $CD4^+$ T, NKT and NK cells in the brains of infected mice.

(A-C) Time course of total cerebral lymphocyte counts during the late stages of blood infection. Mice were infected with WT (blue) or PRF (red) sporozoites by intravenous injection (*n*=6 each), and cerebral lymphocytes were isolated and analyzed at indicated time points. The dotted line represents the mean corresponding cerebral lymphocyte number in all naïve animals (*n*=16). Shown are **(A)** total $CD4^+$ T cell, **(B)** total NKT cell and **(C)** total NK cell numbers. The scatter dot plots represent mean values (± SD). **, P<0.01 (Mann-Whitney test).

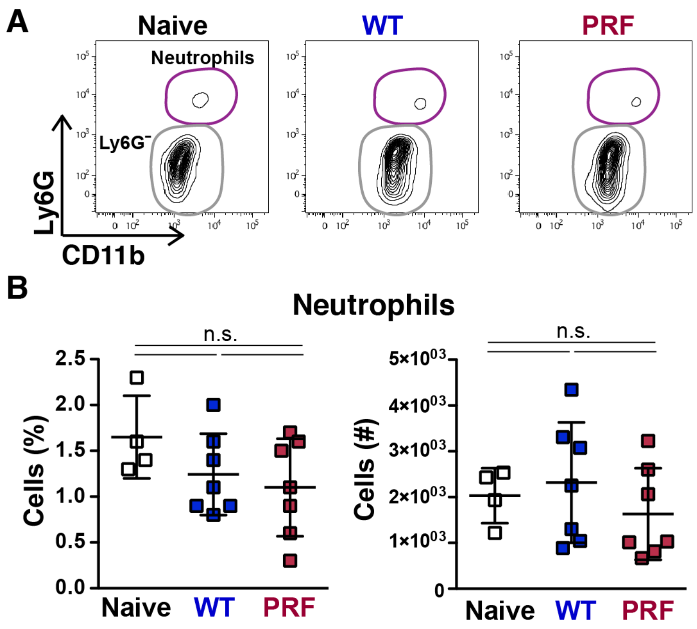

Supplementary Figure 6. Neutrophil infiltration in the brain does not significantly increase on day 8 after sporozoite-induced infection.

(A) Representative contour plots from day 8 after infection showing Ly6G *vs.* CD11b expression on myeloid cells obtained from naïve mice (left), and mice infected with WT (center) and PRF (right) sporozoites. Purple circles indicate gating of neutrophils, $Ly6G^{+}CD45^{med/lo}CD11b^{+}$ cells and grey circles are $Ly6G^{-}$ myeloid cells, $Ly6G^{-}CD45^{med/lo}CD11b^{+}$ cells.

(B) Analysis of neutrophils in the brain. Shown are percentage (left) and numbers (right) of this population in the brain. The scatter dot plots represent mean values (± SD) from samples (n=4-7) isolated 8 days after infection from two independent experiments. Differences were non-significant (Mann-Whitney test).

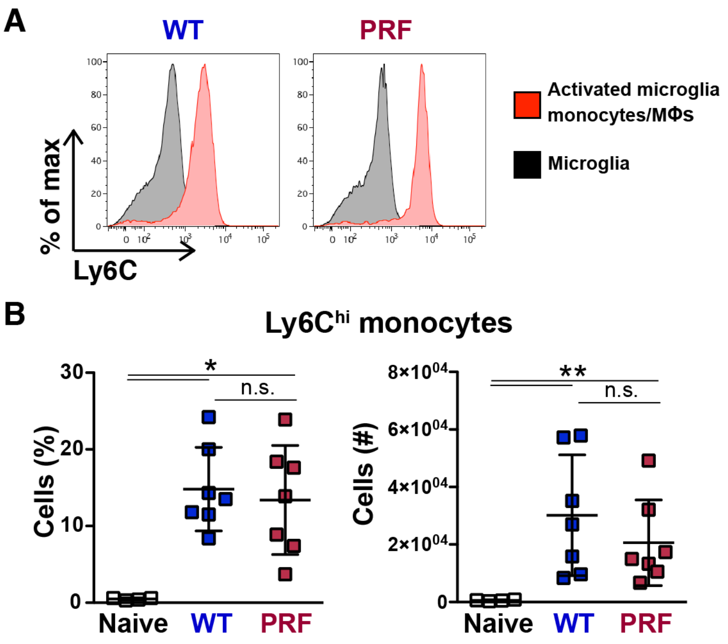

Supplementary Figure 7. Brain-infiltrating myeloid cells are primarily Ly6C^{hi} inflammatory monocytes.

(A) Histogram of Ly6C staining on the Ly6G^{-} myeloid population from mice infected with WT (left) and PRF (right) sporozoites. Gated microglia (grey histogram) display low Ly6C expression; while the gated activated microglia, monocytes and macrophages (red histogram) express high levels of Ly6C.

(B) Quantification of infiltrating Ly6C^{hi} monocytes, defined as Ly6C^{hi} CD45^{med}Ly6G^{-}CD11b^{+} cells. The scatter dot plots represent mean values (± SD) from samples (*n*=4-7) isolated 8 days after infection from two independent experiments. Shown are total percentage (left) and numbers (right) of this cell population in the brain. n.s., non-significant; *, P<0.05; **, P<0.01 (Mann-Whitney test).
